# *Orientocardiochiles*, a new genus of Cardiochilinae (Hymenoptera, Braconidae), with descriptions of two new species from Malaysia and Vietnam

**DOI:** 10.3897/zookeys.971.56571

**Published:** 2020-09-24

**Authors:** Ilgoo Kang, Khuat Dang Long, Michael J. Sharkey, James B. Whitfield, Nathan P. Lord

**Affiliations:** 1 Department of Entomology, Louisiana State University Agricultural Center, 404 Life Sciences Building, Baton Rouge, LA, 70803, USA Louisiana State University Agricultural Center Baton Rouge United States of America; 2 Institute of Ecology & Biological Resources, Vietnam Academy of Science & Technology, 18 Hoang Quoc Viet Road, Cau Giay, Ha Noi, Vietnam Vietnam Academy of Science & Technology Ha Noi Vietnam; 3 Department of Entomology, University of Kentucky, Kentucky, USA University of Kentucky Kentucky United States of America; 4 Department of Entomology, University of Illinois, Urbana, Illinois, USA University of Illinois Urbana United States of America

**Keywords:** Malaysia, morphology, parasitoid wasp, taxonomy, type species, Vietnam

## Abstract

For the first time in 21 years, a new genus of cardiochiline braconid wasp, *Orientocardiochiles* Kang & Long, **gen. nov.** (type species *Orientocardiochiles joeburrowi* Kang, **sp. nov.**), is discovered and described. This genus represents the ninth genus in the Oriental region. Two new species (*O. joeburrowi* Kang, **sp. nov.** and *O. nigrofasciatus* Long, **sp. nov.**) are described and illustrated, and a key to species of the genus, with detailed images, is added. Diagnostic characters of the new genus are analyzed and compared with several other cardiochiline genera to allow the genus to key out properly using an existing generic treatment. The scientific names validated by this paper and morphological data obtained from this project will be utilized and tested in the upcoming genus-level revision of the subfamily based on combined morphological and molecular data.

## Introduction

Cardiochilinae Ashmead, 1900 is a relatively small subfamily of Braconidae Nees, 1811, comprising 221 described species in 16 genera (Yu et al. 2016) and with five of these species recently described (Edmardash et al. 2018; Long et. al. 2019; Dabek et al. 2020). The distribution of the members of the subfamily is cosmopolitan, with the highest diversity in tropical and arid regions (Dangerfield et al. 1999). Members of eight genera are known from the Oriental region, including the introduced genus, *Toxoneuron* (Say, 1836) (Dangerfield et al. 1999). Four cardiochiline braconid genera are known from Malaysia: *Bohayella* Belokobylskij, 1987; *Cardiochiles* Nees, 1819; *Hartemita* Cameron, 1910; and *Psilommiscus* Enderlein, 1912 (Dangerfield et al. 1999). In Vietnam, four genera, *Bohayella*, *Cardiochiles*, *Hartemita*, and *Austrocardiochiles* Dangerfield, Austin & Whitfield, 1999, are reported (Dangerfield et al. 1999; Long and Belokobylskij 2003; Long and van Achterberg 2011a, b, 2014; Long et al. 2019). *Orientocardiochiles* Kang & Long, gen. nov. represents the 17^th^ genus in the world, the ninth genus in the Oriental region, and the fifth genus in both Malaysia and Vietnam.

Females of the subfamily are known as solitary koinobiont endoparasitoids of lepidopteran larvae, laying only one egg in each host and allowing the host to continue its development while parasitized. Larvae of Pyralidae and Noctuidae, among other families, are typically hosts and include important crop pests such as the tobacco budworm, *Heliothis virescens* (Fabricius, 1777), and cotton bollworm, *Heliocoverpa armigera* (Hübner, 1808) (Huddleston and Walker 1988). Of the 13 cardiochiline species of Vietnam’s fauna, one species, *Cardiochiles philippensis* Ashmead, 1905, is reported as an endoparasitoid reared from larvae of the rice leaf-folder, *Cnaphalocrocis medinalis* (Guenée, 1854) (Lepidoptera: Pyralidae) (Long and Belokobylskij 2003).

## Materials and methods

The type specimens for the present work were provided by the Braconidae Collection of the Institute of Ecology and Biological Resources (IEBR: Ha Noi, Vietnam), the Hymenoptera Institute (HIC: 116 Franklin Ave., Redlands, California, USA), and Museums Victoria (MVMA: Melbourne, Victoria, Australia). Other materials were borrowed from HIC and Illinois Natural History Survey (INHS: Champaign, Illinois, USA). All HIC material will be deposited in the Canadian National Collection of Insects (CNC: Ottawa, Ontario, Canada), including the holotype of *Orientocardiochiles joeburrowi* Kang sp. nov. and the holotype of *Orientocardiochiles nigrofasciatus* Long, sp. nov. is housed in IEBR.

The morphological terminology mostly follows Dangerfield et al. (1999) and van Achterberg (1993). Morphological terminology can also be checked at the Hymenoptera Ontology website (http://portal.hymao.org/projects/32/public/ontology/). Terms for sculpture are based on Harris (1979), and wing vein terminology mostly follows the modified Comstock-Needham system (van Achterberg 1993). Definitions of the morphological measurements used in this study are mostly based on van Achterberg (1988).

For the specimen of *O. joeburrowi* sp. nov., morphological analysis was conducted using a Leica MZ75 stereomicroscope. Color habitus images were captured using a Visionary Digital BK Plus imaging system (Dun, Inc.), equipped with a Canon EOS 5DS R DSLR camera. Images were stacked in Zerene Stacker v. 1.04 (Zerene Systems LLC.). All images were made by IK and edited in Adobe Photoshop CS 6 (Adobe Systems, Inc). Body parts of the specimen were also measured using Adobe Photoshop CS 6 (Adobe Systems, Inc).

For the specimen of *O. nigrofasciatus* sp. nov., morphological analysis was conducted using an Olympus SZ61 binocular microscope; measurement were carried out using an Olympus SZ40 binocular microscope; the photographs were produced by KDL with a Sony 5000 digital camera attached to a Nikon SMZ 800N binocular microscope at IEBR and processed with Adobe Photoshop CS5 to adjust the size and background.

Abbreviations used in this paper are as follows: POL: distance between posterior ocelli; OOL: distance between posterior ocelli and eye; OD: diameter of posterior ocellus; T1: first metasomal tergum; T2: second metasomal tergum; T3: third metasomal tergum; MT: Malaise trap; “Card. + number”: code number indexing for specimens of the Cardiochilinae in the collection at IEBR; NP: National Park, S: South.

The key to species of *Orientocardiochiles* gen. nov. and descriptions of the two species are based on females. Distribution maps were produced using QGIS 3.10.0 (QGIS Development Team 2019). Google satellite maps were downloaded via the QuickMapServices plugin.

## Results

### Taxonomy

#### Orientocardiochiles

Taxon classificationAnimaliaHymenopteraBraconidae

Kang & Long gen. nov.

A5DC33EC-BD78-503D-A3B1-A1C514A89E4A

http://zoobank.org/B3CD416F-0231-46D0-92F9-9EDD6A6B7204

##### Type species.

*Orientocardiochiles joeburrowi* Kang, sp. nov.

##### Diagnosis

(based on all the members of the genus). Body large and stout, finely sculptured, whitish to yellow pale in color with black spots and stripes. Head in dorsal view transverse. Antenna 41- or 43-segmented. Eyes sparsely pilose. Clypeus with distinct suture and two clypeal tubercles present apically. Malar suture present. Mandible bidentate and angularly bent ventrally. Mouthparts (the length of galea and glossa) short. Maxillary palpus 5- or 6-segmented. Labial palpi 4-segmented. Notauli deep, crenulate, meeting posteriorly in deep smooth area. Scutellar sulcus curved, with 5+ crenulae. Scutellum more or less elevated medially, without carina laterally and apically. Propodeal areola completely developed and kite-shaped or elongated pentagonal. Epicnemial carina absent. Mesopleuron mostly smooth; precoxal sulcus well-defined and crenulate, not reaching posterior margin. Metapleuron rugulose. Mesosternal sulcus finely crenulate. Hind tibia without apical projection; inner tibial spur distinctly longer than outside spur, subequal to half of hind basitarsus. Tarsal claws pectinate. Forewing with elongated pterostigma; vein r reaching at apical fourth of pterostigma; SR1 sharply angled at basal fourth; basal fourth of vein SR1 almost perpendicular to apical vein 3-SR. Vein 1a present as a spectral short trace; 1^st^ discal cell in forewing rather short compared to first submarginal cell. Second submarginal cell elongated. First subdiscal (brachial) cell broad. M+CU in hind wing distinctly shorter than 1-M. Hind wing with 6 hamuli. T1 widened apically, with lateral suture clearly defined throughout. T2 mostly rugose except for plateau-like projection (Figs 2D, 5G); plateau-like projection of T2 present at anteromedial base. T3 entirely smooth. Hypopygium sharply pointed at apex, median longitudinal area evenly sclerotized or largely desclerotized medially throughout; median enfold of hypopygium present or absent. Ovipositor sheath longer than metasoma, pointed at apex, and with short setae throughout.

##### Distribution.

Oriental (Malaysia, Vietnam).

##### Biology.

Unknown.

##### Etymology.

The name for the genus refers to *Cardiochiles* from the Oriental region. From “orientum” (Latin for the eastern region) and the generic name “*Cardiochiles* Nees, 1819.” Gender: masculine.

##### Notes.

*Orientocardiochiles* gen. nov. will run to couplet 9 in the key to world genera by Dangerfield et al. (1999), but it can be distinguished from *Austerocardiochiles* in the couplet 9b of the key as follows:

**Table:** 

9b	Eyes densely setose (Fig. 1F); mandible evenly curved ventrally (Fig. 1F); body well-sculptured; scutellum well-punctate, more or less depressed medio-posteriorly and with carina; epicnemial carina often present; vein M+CU of hind wing slightly longer or subequal to vein 1-M; submedian field of second metasomal tergite lens-shaped (Fig. 1D); hypopygium relatively short and obtuse apically (Fig. 1A, C); ovipositor sheath widened apically, shorter than metasoma (Fig. 1C)	***Austerocardiochiles* Dangerfield, Austin & Whitfield, 1999**
–	Eyes sparsely setose (Fig. 2F); mandible angularly bent ventrally (Figs 2F, 5C); epicnemial carina absent; vein M+CU of hind wing distinctly longer than vein 1-M; plateau-like projection on second metasomal tergum (Fig. 2D; hypopygium strongly elongated and acute apically (Figs 2A, 2C, 5N); ovipositor sheath not widened apically (Fig. 5O), distinctly longer than metasoma (Figs 2C, 5A)	***Orientocardiochiles* Kang & Long, gen. nov.**

### Key to species of the genus *Orientocardiochiles* Kang & Long, gen. nov.

**Table:** 

1	Forewing entirely lightly infuscate (Fig. 2A, B); propodeum without short basal carina and propodeal areola quadrate (kite-shaped) (Fig. 2D); hind tarsal claw pectinate with 10 teeth (Fig. 2G); scape entirely brown (Fig. 2F)	***O. joeburrowi* sp. nov.**
–	Forewing antero-apically strongly infuscate (Fig. 5H); propodeum with short basal carina and propodeal areola elongate pentagonal (Fig. 5J); hind tarsal claw pectinate with four teeth (Fig. 5L); scape mostly yellow (Fig. 5A)	***O. nigrofasciatus* sp. nov.**

**Figure 1.**
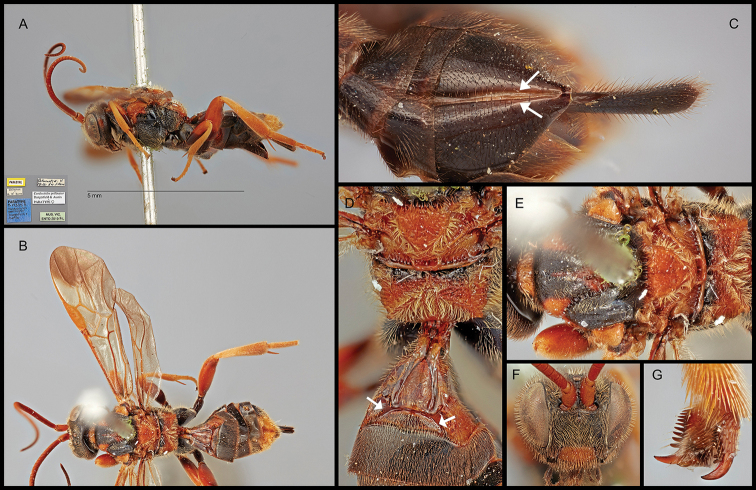
*Austerocardiochiles pollinator*, paratype. **A** Lateral habitus **B** dorsal habitus **C** ventral metasoma; arrow: median enfold on hypopygium **D** dorsal propodeum and metasomal terga 1 to 3; arrow: lens-shaped area **E** dorsal mesonotum **F** anterior head **G** claws.

### Species descriptions

#### Orientocardiochiles joeburrowi

Taxon classificationAnimaliaHymenopteraBraconidae

Kang sp. nov.

C6C0A498-FC35-580B-A693-6E227D16571E

http://zoobank.org/C653D411-AED6-45FA-8BE7-254DBDA02BD9

Fig. 2A–G

##### Material examined.

***Holotype*** Malaysia • ♀; female, Perlis, Wang Kelian; 6°40'40.94"N, 100°11'23.94"E; xi.2008; Sharkey & Norliyana.

##### Description.

**Figure 2.**
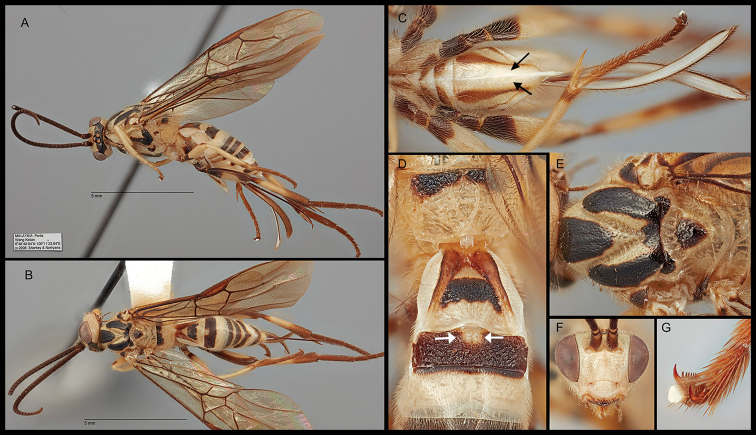
*Orientocardiochiles joeburrowi* sp. nov., holotype. **A** Lateral habitus **B** dorsal habitus **C** ventral metasoma; arrow: hypopygium **D** dorsal propodeum and metasomal terga 1–3; arrow: plateau-like projection **E** dorsal mesonotum **F** anterior head **G** claws.

Body large and stout, 9.1mm. Antenna 6.4 mm. Length of forewing 9.6 mm. Ovipositor sheath 4.4 mm. ***Head*.** Antenna 41-segmented; length of scape 1.3 × longer than its width (30:23); third segment (basal flagellomere) 2.2 × longer than second segment (pedicel) (29:13); apical segment 1.9 × longer than subapical segment (15:8). Clypeal suture distinct (Fig. 2F); with two well-developed tubercles; width of clypeus 1.9 × its height (72:44); face width 0.9 × length of face and clypeus combined (11:12); distance between tentorial pits 1.9 × distance between a pit and eye margin (60:32). Mandible bidentate; basal width of mandible 0.7 × longer than the distance from mandible to eye margin (22:31). Maxillary palpus 5-segmented. Labial palpus 4-segmented. Galea short with dense setae (Fig. 2F). Glossa short (Fig. 2F). Head transverse, median length 0.35 × longer than the maximum width of head in dorsal view (75:217). Eye length 2.0 × length of temple as viewed dorsally (72:36). Ocellar triangle marginated with shallow suture; POL:OD:OOL= 10:18:42. ***Mesosoma*.** Length of mesosoma 1.4 × its height (37:26). Notauli present (Figs 2B, E). Mesoscutum with shallow submarginal furrows (Fig. 2E). Scutellar sulcus curved with 5 crenulae, 0.33 × longer than median length of scutellum (19:57) (Fig. 2E). Postscutellar depression absent. Propodeum rugulose; propodeal areola kite-shaped, length of median areola 1.8 × longer than its maximum width (60:34); median transverse carina on the propodeum reaching lateral margin (Fig. 2D). Pronotum mostly smooth and carinate posteriorly. Mesopleuron mostly smooth; precoxal sulcus well-defined and crenulate, not reaching posterior margin. Metapleuron rugulose. Mesosternal sulcus with few barely perceptible crenulae. ***Legs*.** Fore tibial spur 0.57 × basitarsus (44:77). Length of hind femur, tibia and basitarsus 3.8× (210:55), 7.1× (320:45) and 6.0 × (18:3) longer than maximum width of each. Basal spur of mid tibia 0.58× longer than length of mid-basitarsus (67:115). Basal spur of hind tibia 1.8 × longer than length of apical spur (88:49), and 0.49 × longer than length of hind basitarsus (88:178). Hind basitarsus 0.56 × longer than length of hind tibia (18:32), and 0.96 × longer than length of remaining hind tarsi 2–5 (178:185). Hind tarsal claws pectinate with 10 teeth (Fig. 2G). ***Wings*.** Length of forewing 3.2 × longer than its maximum width (96:32). Length of pterostigma 4.4 × longer than its width (191:44). Forewing r:3-SR:2-SR= 33:165:99; 1-M 2.4 × longer than m-cu (88:36); 2-SR+M 1.63 × longer than m-cu (59:36); 1-CU1 0.23 × longer than 2-CU1 (22:96) and 0.37 × longer than cu-a (22:59). Length of hind wing 5.2 × longer than its maximum width (78:15); second submarginal cell trapezoid, maximum length of the cell 3.15× longer than its maximum height (262:83) (Fig. 2A). Hind wing M+CU distinctly shorter than 1-M, and 0.63 × longer than 1-M (75:119); 1-M 3.6 × longer than length of 1r-m (119:33); 2-SC+R horizontal to the longitudinal axis of hind wing; 2-1A absent. ***Metasoma*.** T1 punctate medially, about 1.1× longer than its apical width (133:125). (Fig. 2D). T2 dorsally rectangular; median length of T2 0.34 × longer than its apical width (50:146), and 0.74 × as long as median length of T3 (50:67) (Figs 2B, D). T3 entirely smooth (Fig. 2D). Hypopygium acute apically, fully sclerotized without median suture (Fig. 2A, C). Ovipositor length about 1.23× longer than length of metasoma (57:46). Ovipositor sheaths densely setose throughout; setose part of ovipositor sheath 0.95 × longer than length of metasoma (44:46), 1.38 × longer than length of hind tibia (44:32), and 0.46 × longer than length of forewing (44:96).

***Color*.** Body mostly whitish pale and appearing striped; the following areas melanic: antenna, vertex, median mesonotal lobe (mostly melanic except for posterior area), lateral mesonotal lobe (pale basally), scutellum, anterior propodeum, fore trochantellus, basal fore femur, mid trochanter (mostly) and trochantellus, hind coxa with a large melanic spot posterolaterally, entire hind trochanter and trochantellus, hind femur (except for anteromedial area), mid and hind tarsi, median tergum 1, entire tergum 2, anterior terga 3–6, posterior tergum 7, ovipositor and external ovipositor sheaths. Wings entirely lightly infuscate, stigma dark brown but centrally pale.

**Male.** Unknown.

##### Etymology.

Named in honor of Joseph Lee Burrow, the world-class college football quarterback for the LSU Tigers and the 2019 Heisman Trophy winner.

##### Host(s).

Unknown.

**Figure 3.**
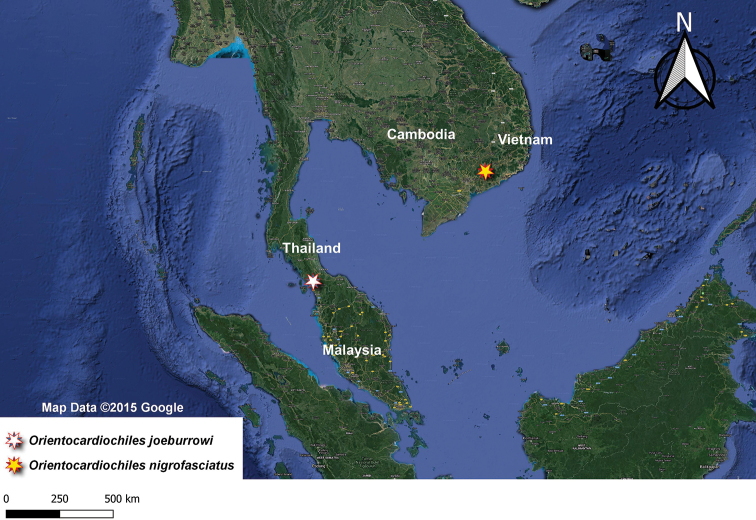
Distribution map of the members of *Orientocardiochiles* gen. nov. in Malaysia and Vietnam.

##### Distribution.

**Figure 4.**
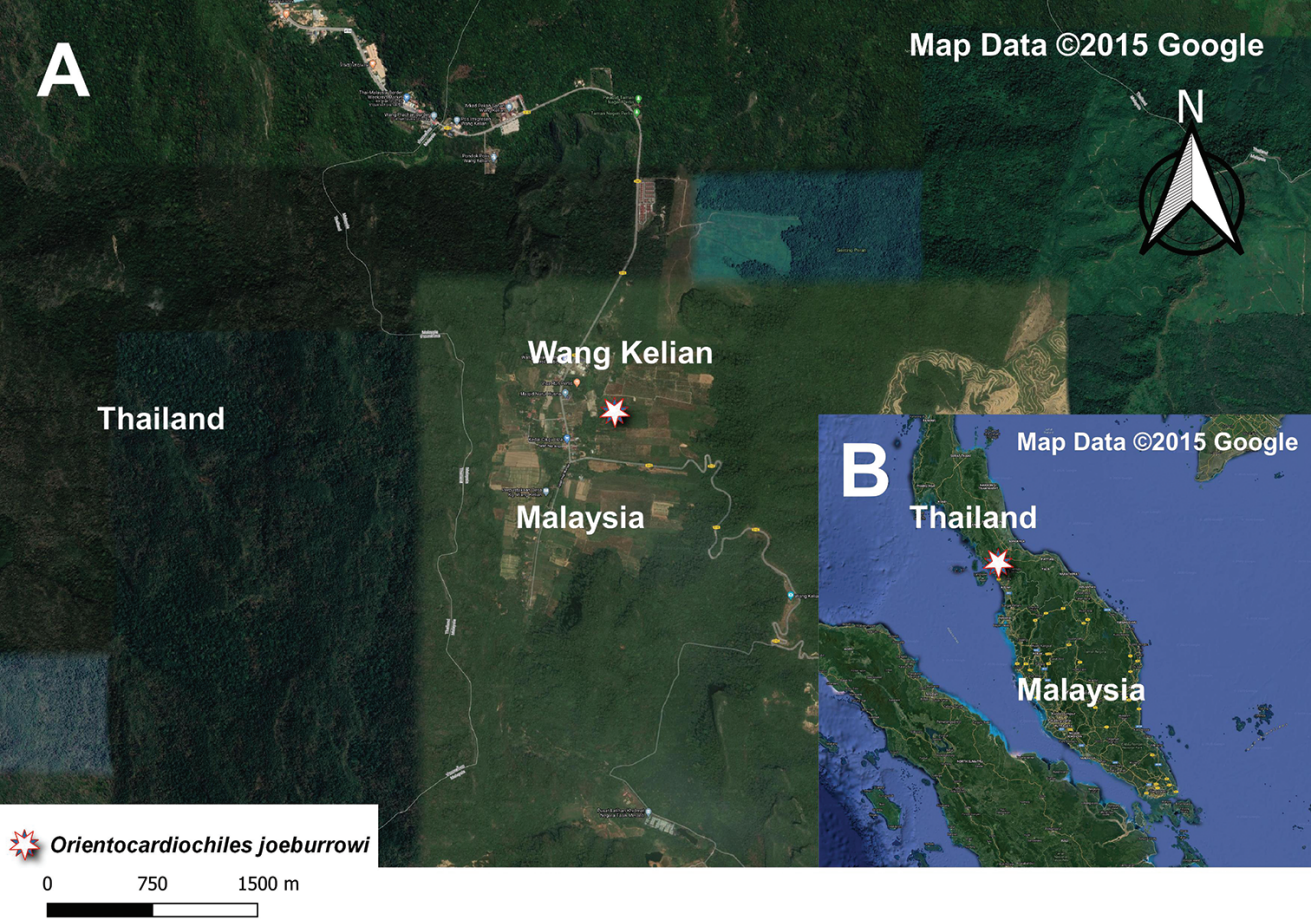
**A** Distribution map of *Orientocardiochiles joeburrowi* sp. nov. in Wang Kelian **B** distribution map of *O. joeburrowi* sp. nov. in Malaysia.

*Orientocardiochiles joeburrowi* sp. nov., is known from only one female specimen collected from Wang Kelian, Malaysia, which is near the Thailand–Malaysia border (Fig. 4A, B).

##### Notes.

*Orientocardiochiles joeburrowi* sp. nov., can be distinguished from *O. nigrofasciatus* sp. nov. due to the following diagnostic characters of the genus: i) forewing entirely lightly infuscate; ii) propodeum without short longitudinal carina anteriorly; iii) propodeal areola quadrate (kite-shaped); iv) hind tarsal claw pectinate with 10 teeth; v) hypopygium entirely sclerotized and without median enfold; vi) scapus entirely brown.

#### Orientocardiochiles nigrofasciatus

Taxon classificationAnimaliaHymenopteraBraconidae

Long sp. nov.

7F1AAC72-BFDB-594B-8501-81F5A2662298

http://zoobank.org/7F9CC61E-28E0-45A2-B6C4-40FA9F4231A9

Fig. 5A–O

##### Material examined.

***Holotype***, Vietnam • ♀; female, “**Card.101**” (IEBR), S. Vietnam: Lam Dong, Cat Tien NP, forest; 11°18'N, 107°26'E, 100 m; 8.iv.2007; MP Quy.

##### Description.

**Figure 5.**
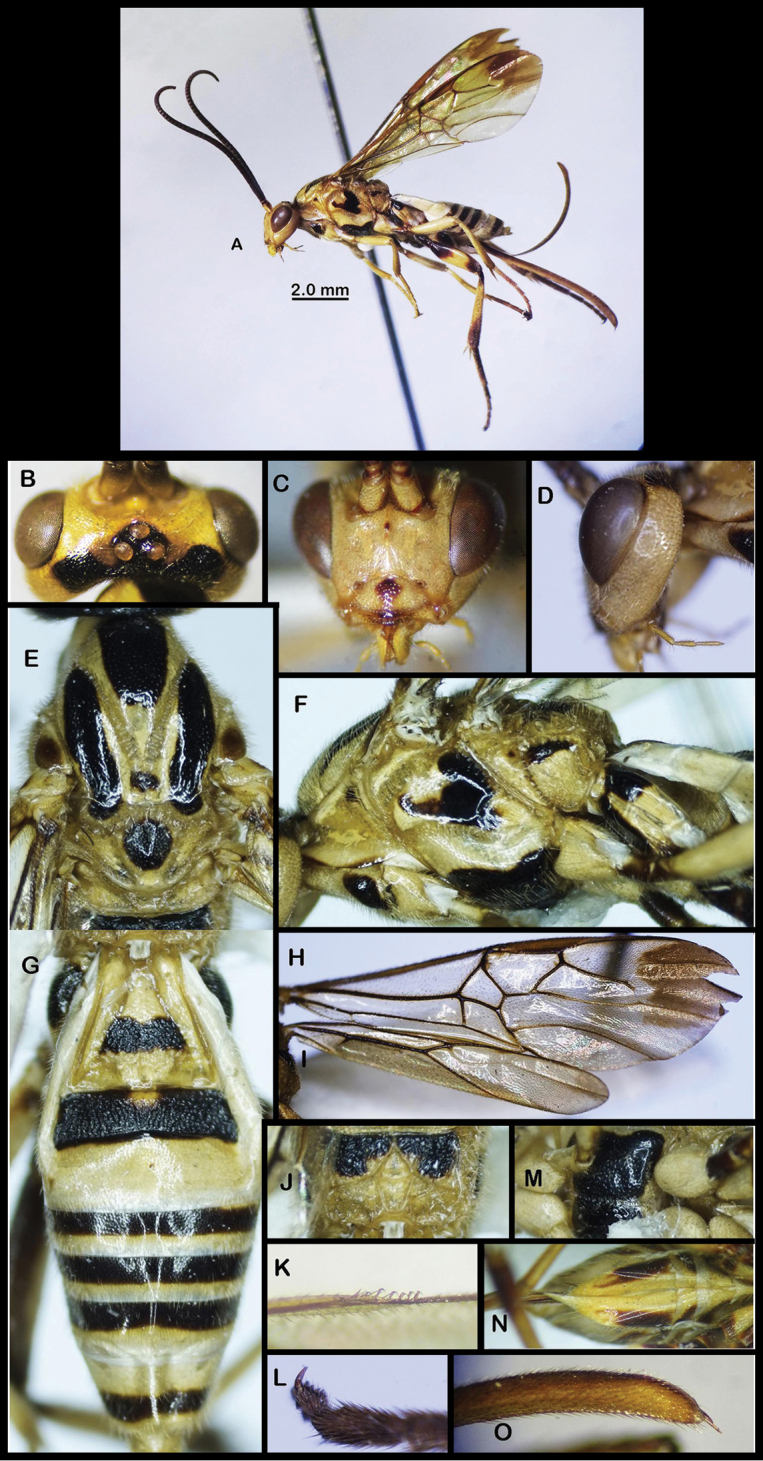
*Orientocardiochiles nigrofasciatus* sp. nov. **A** Lateral habitus (holotype, female) **B** head (dorsal view) **C** head (anterior view) **D** head (lateral view) **E** dorsal mesonotum **F** lateral mesonotum **G** metasoma (dorsal view) **H** forewing **I** hind wing **J** propodeum **K** hamuli on hind wing **L** hind tarsal claw **M** ventro-lateral mesonotum **N** ventral metasoma **O** apex of ovipositor sheath (lateral view).

Body length 9.7 mm. Length of forewing 9.0 mm. Antenna 7.0 mm, ovipositor sheath 4.7 mm. ***Head*.** Antenna with 43 segments; length of scape 1.4 × longer than its width (18:13); third segment 1.5 × longer than second segment (15:10); apical segment 2.25 × longer than subapical segment (4:10). Clypeal suture distinct; ventral margin of clypeus evenly convex with indistinct tubercles (Fig. 5C); width of clypeus 1.8 × longer than its height (35:19); face width 0.9 × length of face and clypeus combined (28:32); distance between tentorial pits 1.9 × distance between pit and eye margin (15:8) (Fig. 5C). Mandible angularly bent ventrally (Fig. 5C); basal width of mandible 0.8 × distance from mandible to eye margin (8:10). Frons depressed laterally, with tubercle anteriorly, almost smooth; in dorsal view. Head transverse; median length of head 0.45 × its width (25:56) in dorsal view. Eye length 1.9 × length of temple (17:9). Ocelli rather large; POL:OD:OOL=3:4:13 (Fig. 5B). Vertex sparsely punctate anteriorly, rugose-punctate posteriorly; in lateral view. Length of eye 1.3 × temple (18:14); temple sparsely punctate (Fig. 5D). ***Mesosoma*.** Mesosoma robust; length of mesosoma 1.6 × height (55:35) (Fig. 5F). Pronotal side large, almost smooth. Notauli evenly deep, crenulate, meeting deep smooth area posteriorly (Fig. 5E). Lobes of mesoscutum shiny, sparsely punctate. Scutellum slightly convex medially, densely and finely punctate. Scutellar sulcus rather narrow, curved, with 5+ crenulae, median length of scutellar sulcus 0.3 × longer than median length of scutellum (7:24) (Fig. 5E). Propodeal areola length 1.8 × longer than its width (27:15). Epicnemial carina absent (Fig. 5M). Precoxal sulcus wide, shallow, crenulate (Fig. 5F). Mesopleuron sparsely and finely punctate. Subalar space crenulate. Metapleuron smooth anteriorly, foveate anteriorly. Propodeum with short basal carina; propodeal areola complete, almost occupying whole length of propodeum, areola with two median transverse carinae (Fig. 5J); propodeum coarsely rugose laterobasally. ***Legs*.** Fore tibial spur 0.6 × longer than basitarsus (21:35). Length of hind femur, tibia and basitarsus 3.0, 7.0 and 8.0 × longer than their maximum width, respectively. Hind coxa shiny, smooth. Hind femur sparsely punctate. Hind tibia without apical projection; inner hind tibial spur 1.6 × longer than outer spur (16:10) and 0.5 × longer than hind basitarsus (16:32). Hind basitarsus 0.5 × longer than hind tibia (32:63), 0.9 × longer than hind tarsus 2–5 (32:37). Hind tarsal claw pectinate, with 4 teeth (Fig. 5L). ***Wings*.** Length of forewing 3.1 × longer than its maximum width (90:29). Pterostigma elongate; length of pterostigma 5.0 × longer than its width (45:9) (Fig. 5H). r:3-SR:2-SR=6:33:18. 1-M 2.4 × as long as m-cu (17:7). 2-SR+M 1.7 × as long as m-cu (22:13). 1-CU1 0.14 × 2-CU1 (4:29) and 0.22 × cu-a (4:18). 1a present as a spectral short trace. Second submarginal cell long, maximum length 3.1 × longer than its maximum width (90:29) (Fig. 5H). Subdiscal cell broad. Length of hind wing 5.3 × longer than its maximum width (101:19). M+CU of hind wing distinctly shorter 1-M, and 0.65 × longer than 1-M (15:23). 1-M 4.6 × 1r-m (23:5). 2-SC+R horizontal to the longitudinal axis of hind wing (Fig. 5I). Hind wing with six hamuli (Fig. 5K). ***Metasoma*.** T1 widened apically, 0.96 × longer than it is wide (28:29) (Fig. 5G); coriaceous smooth basally, almost punctate-reticulate medially, rugose apically. T2 transverse, without emarginate basal area, largely rugose (Fig. 5G); median length of T2 0.3 × longer than its apical width (10:32), and 0.6 × longer than median length of T3 (10:16). T3 sparsely and finely punctate. Remaining tergites almost smooth (Fig. 5G). Hypopygium sharply pointed at apex, median longitudinal area largely desclerotized and folded inwards throughout (Fig. 5N). Ovipositor sheath slender, pointed at apex and shortly setose (Fig. 5O); setose part of ovipositor sheath 1.4 × longer than length of metasoma (64:47), 2.0 × longer than length of hind tibia (64:32), and 0.7 × longer than length of forewing (64:90).

***Color*.** Pale yellow; antenna black, except scape yellow; stemmaticum and vertex posteriorly black (Fig. 5B); lobes of mesoscutum largely black medially; scutellum black, pale yellow laterally and apically (Fig. 5E); propodeum black medio-basally (Fig. 5J); propleuron posteriorly, mesopleuron medio-dorsally and mesosternum black (Fig. 5F); fore and middle legs pale yellow, except middle trochanters, trochantellus, and tarsus yellowish-brown; hind coxa dorso-basally and ventrally, trochanters, trochantellus, femur basally and apically, hind tibia at base and apically, hind tarsus brown; wing veins brown; wing membrane hyaline, apex of forewing blackish-brown (Fig. 5H); first metasomal tergite with large median black patch; second tergite black, except basal small round yellow area; third tergite pale yellow; fourth–sixth tergites with basal black stripes (Fig. 5G); seventh tergite black apically.

**Male.** Unknown.

##### Etymology.

From “nigro” (Latin for “black”), and “fascia” (Latin for “band”, “zone”, “stripe”), because of black stripes basally on metasomal tergites 4–6.

##### Host(s).

Unknown.

##### Distribution.

**Figure 6.**
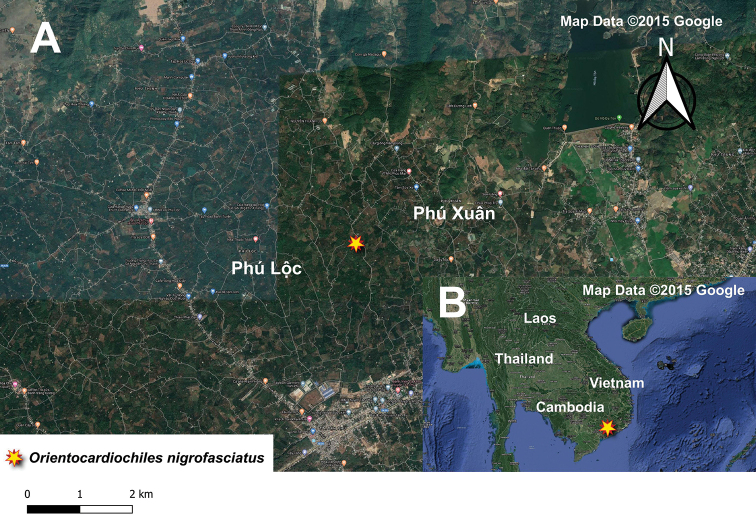
**A** Distribution map of *Orientocardiochiles nigrofasciatus* sp. nov. in Cat Tien NP **B** distribution map of *O. nigrofasciatus* sp. nov. in Vietnam.

*Orientocardiochiles nigrofasciatus* sp. nov., is known from only one female specimen collected from Cat Tien NP, S. Vietnam. (Fig. 6A, B).

##### Notes.

*Orientocardiochiles nigrofasciatus* sp. nov., from Vietnam can be separated from *Orientocardiochiles joeburrowi* sp. nov., from Malaysia by the following characters: i) forewing apically strongly infuscate; ii) propodeum with short longitudinal carina anteriorly; iii) propodeal areola pentagonal; iv) hind tarsal claw pectinate with four teeth; v) hypopygium with median longitudinal fold; vi) scape mostly yellow, except for the dorso-apical region.

## Discussion

### Character discussion

Members of *Orientocardiochiles* Kang & Long, gen. nov., *Austerocardiochiles* Dangerfield, Austin, & Whitfield, 1999, *Hansonia* Dangerfield, 1996, *Heteropteron* Brullé, 1846, and *Wesmaelella* Spinola, 1851 share the presence of lateral sutures on the first metasomal tergum that are clearly defined throughout the length of the tergum (Figs 1D, 2D, 5G). According to the most recent complete genus-level phylogeny of the subfamily based on morphological characters (Dangerfield et al. 1999), three monophyletic clades are separated based on the presence or absence and length of eye setae.

**Figure 7.**
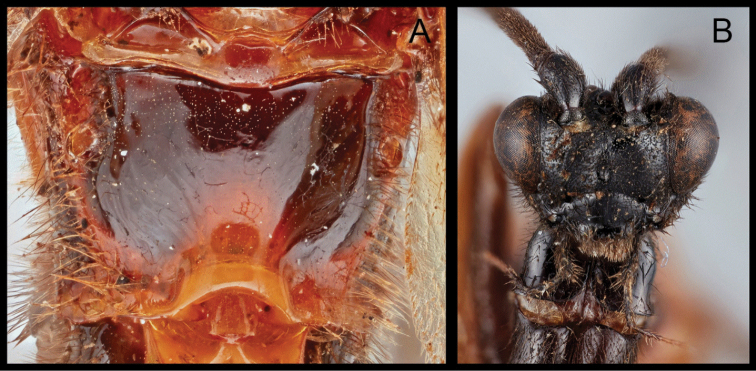
*Heteropteron* sp. **A** Dorsal propodeum **B** anterior head.

The basal clade of Dangerfield et al. (1999) is composed of members of *Heteropteron* and *Wesmaelella* that have glabrous eyes (Fig. 7B). The members of these genera do not possess clypeal tubercles, nor a median areola on propodeum (Fig. 7A, B). Also absent is the lens-shaped area, or plateau-like projection on the second metasomal tergum. Based on the characters mentioned, *Orientocardiochiles* gen. nov. cannot be placed in the basal clade.

Clade A of Dangerfield et al. (1999) contains *Hansonia* and other genera that have short and sparse eye setae as in *Orientocardiochiles* gen. nov. (Figs 2F, 5C, D). Members of *Austerocardiochiles* and other genera in clade B mostly have long and dense eye setae (Fig. 1F), unlike *Orientocardiochiles* gen. nov. Based on the eye setae character, *Orientocardiochiles* gen. nov. can be placed in clade A. However, *Orientocardiochiles* gen. nov. could be included in the clade B if the short eye setae character is an independently developed character, as has occurred in one of the basal genera of the clade B, *Asiacardiochiles* Telenga, 1955. Based on the hypopygial median fold of *O. nigrofasciatus* sp. nov. (Fig. 5N), placing *Orientocardiochiles* gen. nov. in clade B is more probable than clade A because members of two genera in clade B, *Austerocardiochiles* and *Cardiochiles*, possess the hypopygial median fold (Fig. 1C).

### Distribution and diversity

As mentioned in the Introduction, members of Cardiochilinae are distributed worldwide. Regarding the genus-level diversity, the Australasian region has the highest diversity. Ten cardiochiline genera have been recorded from the Australasian region (Dangerfield et al. 1999; Yu et al. 2016). As a result of this work, we confirmed that Cardiochilinae exhibits the second highest genus-level diversity in the Oriental region. Nine genera, including the introduced genus *Toxoneuron* (Say, 1836), are recorded from the region. Five genera have been recorded from Malaysia and Vietnam in each country. More genera will be likely to be found in Malaysia and Vietnam after further collecting.

### Future directions

In the past two decades, molecular data combined with morphological data has been widely utilized to improve resolution of the species-, genus-, tribe-, and subfamily-level relationships of Braconidae. However, a genus-level phylogeny of Cardiochilinae based on molecular data is lacking. This shows the necessity of a novel phylogeny. Therefore, IK is in process of conducting a new genus-level revision of Cardiochilinae to elucidate the genus-level relationships of the subfamily using a novel phylogeny based on combined molecular and morphological data.

## Supplementary Material

XML Treatment for Orientocardiochiles

XML Treatment for Orientocardiochiles joeburrowi

XML Treatment for Orientocardiochiles nigrofasciatus
